# Detection of lymph node metastasis in colon cancer by ectopically expressed fibroblast markers FOXQ1 and THBS2

**DOI:** 10.3389/fonc.2023.1297324

**Published:** 2023-12-14

**Authors:** Haytham Ali, Manar AbdelMageed, Lina Olsson, Gudrun Lindmark, Marie-Louise Hammarström, Sten Hammarström, Basel Sitohy

**Affiliations:** ^1^ Department of Clinical Microbiology, Umeå University, Umeå, Sweden; ^2^ Department of Radiation Sciences, Oncology, Umeå University, Umeå, Sweden; ^3^ Department of Animal and Veterinary Sciences, College of Agricultural and Marine Sciences, Sultan Qaboos University, Muscat, Oman; ^4^ Department of Pathology, Faculty of Veterinary Medicine, Zagazig University, Zagazig, Egypt; ^5^ Institution of Clinical Sciences, Lund University, Lund, Sweden

**Keywords:** THBS2, FOXQ1, MMP11, CXCL12, colon cancer, fibroblasts, qRT-PCR, immunohistochemistry

## Abstract

**Introduction:**

Approximately 25% of colon cancer (CC) patients having curative surgery will relapse. Therefore, it is crucial to identify patients with increased recurrence risk to offer them adjuvant chemotherapy. Three markers with prominent expression in fibroblasts: forkhead box Q1 (FOXQ1), matrix metalloproteinase-11 (MMP11), and thrombospondin-2 (THBS2), and the fibroblast expressed chemokine CXCL12 were selected for studies because of the critical role of fibroblasts in the microenvironment of the tumor.

**Methods:**

The expression levels of the biomarkers were assessed in primary CC tumors, lymph nodes of CC patients and controls, and CC cell lines at mRNA and protein levels by real-time qRT-PCR and immunohistochemistry, respectively.

**Results:**

FOXQ1, MMP11, and THBS2 mRNAs were expressed at significantly higher levels in primary tumors compared to normal colon (*P*=0.002, *P*<0.0001, and *P*<0.0001, respectively). In contrast, CXCL12 mRNA levels were higher in normal colon tissue. FOXQ1, MMP11, and THBS2 levels were also expressed at significantly higher levels in metastasis-positive lymph nodes compared to both metastasis-negative- and control nodes *(P*<0.0001*/P*=0.002, *P*<0.0001/P<0.0001, and *P*<0.0001/P<0.0001, respectively). Immuno-morphometry revealed that 30–40% of the tumor cells expressed FOXQ1, MMP11, and THBS2. FOXQ1 and THBS2 were barely detected in normal colon epithelium (*P*<0.0001), while MMP11 was expressed in normal colon epithelium at high levels.

**Discussion:**

We conclude that CC tumor cells show ectopic expression of FOXQ1 and THBS2 possibly making these tumor cells independent of fibroblast cell support. The high expression levels of these two biomarkers in metastatic lymph nodes suggest that they are potential indicators of patients at risk for recurrence.

## Introduction

Colon cancer (CC) is a leading cause of cancer-related deaths worldwide, with an incidence rate of 6% and a mortality rate of 5.8% (1). Surgery is the main treatment modality for CC. However, approximately 25% of CC patients having curative surgery will relapse of which the majority will die (2, 3). There is a need to develop methods that can identify those patients that would benefit from adjuvant chemotherapy treatment (ACT). One avenue that appears very promising is to identify biomarkers that are expressed in CC cells and measure the mRNA levels of these biomarkers in metastatic lymph nodes (LNs), rather than in the primary tumor. Theoretically one would assume that a biomarker that is overexpressed in LN metastases is important for the cancer cells and their survival and development to distant metastases. Our aim is to identify such biomarkers. In a previous study, a genome wide screening was used to compare gene expression levels in 4 metastatic LNs of 4 tumor-node-metastasis (TNM)-stage III patients and the primary tumor of 3 of these patients, with a control group consisting of LNs from 3 patients with inflammatory bowel disease and 1 colon lipoma patient, and 3 samples of normal colon epithelial cells. Eighteen genes were expressed >5-fold higher levels in all cancer samples (4). Previously, KLK6 was selected from this list and was found to have high prognostic value in predicting recurrence in colorectal cancer (CRC). Three of the genes on the list showed prominent expression in fibroblasts and were selected in this study because of the established importance of fibroblasts in the microenvironment of the tumor (5, 6). They were forkhead box Q1 (FOXQ1), matrix metalloproteinase-11 (MMP11) and thrombospondin-2 (THBS2). Moreover, they were upregulated in primary CC tumors according to the Human Protein Atlas (7). For comparative purposes, the stromal cell derived chemokine CXCL12 was also investigated and we have previously shown that the chemokines CXCL16 and CXCL17 are ectopically expressed in primary CC tumors and LN metastasis (8, 9).

FOXQ1 is a transcription factor that regulates the expression of genes involved in cell proliferation and differentiation (10). It has been shown to be involved in activating the oncogenic Wnt/β-catenin signaling pathway (10). FOXQ1 expression was increased in CC tissues and its overexpression was attributed with resistance of CC cells to radiation (11). Moreover, FOXQ1 was shown to induce invasion and metastasis via activation of the HB-EGF/EGFR pathway (12). MMP11 is a zinc-dependent proteolytic metalloenzyme and a member of the matrix metalloproteinase family with more than 20 members in humans (13). MMP11 is activated intracellularly by another protease, furin, promoting the signal transduction of IGF1/protein kinase B (AKT)/FOXO1, which is associated with the metabolic transformation of tumors (14). MMP11 is produced by peritumoral stromal fibroblasts, it regulates early tumor invasion, implantation and expansion and it prevents apoptosis of early cancer cells (13, 15). Recently, high expression levels of MMP11 in CRC primary tumors compared to normal colon tissue was demonstrated (16). Paradoxically, MMP11 has also been suggested to act as a tumor suppressor by inhibiting the metastasis of some tumors (13). THBS2 is an adhesive glycoprotein that mediates cell-to-cell and cell-to-extracellular matrix interactions. It is a ligand to CD36 mediating anti-angiogenic properties (17). THBS2 expression was correlated with immune cell infiltrates in CRC and bad prognosis (18, 19). THBS2 exerted promotional effects on CRC cell proliferation, invasion, and migration, partly by modulating the Wnt/β-catenin signaling pathway (20). CXCL12, also known as stromal cell-derived factor-1 (SDF-1), is a homeostatic chemokine originally reported to regulate hematopoietic cell trafficking and secondary lymphoid tissue architecture (21). It is a ligand for CXCR4. Activation of CXCL12-CXCR4 axis was observed to enhance chemotaxis, trans-endothelial migration, angiogenesis and invasion and metastasis (22, 23). Its high expression in CRC was reported to be associated with bad prognosis (24, 25).

Here we compare the expression of the four fibroblast biomarkers in primary CC tumor tissue and in LNs of CC patients both at the mRNA and protein levels. To investigate the expression pattern of the biomarkers and verify their expression in fibroblasts a selected group of cell lines were also investigated. We found that FOXQ1, MMP11, and THBS2 levels were significantly higher in primary CC tumors and metastasis-positive LNs compared to controls and that FOXQ1 and THBS2 were ectopically expressed.

## Materials and methods

### Patients and tissue specimens for mRNA analysis

Primary tumor specimens were collected from 32 CC patients after surgery at Norrland University Hospital, Umeå, Sweden and Helsingborg Hospital, Helsingborg, Sweden from 2004 to 2016. None of the patients received treatment before surgery. Normal colon samples were retrieved from the distal resection margin of 30 CC patients. LNs were retrieved from 28 CC patients and control LNs from 10 patients, eight with ulcerative colitis, one with Crohn’s disease, and one with lipoma. Gender, age, TMN-stage and tumor location of the patients donating tissues for mRNA analysis, is shown in **Table 1**. Each LN was bisected, where one half was subjected to routine histopathology examination, that is, processed for hematoxylin-eosin (H&E) staining and inspection by specialized pathologists. The other half was snap frozen and kept at –70°C until RNA extraction.

**Table 1:** Clinical characteristics of CC and control patients who donated primary tumor tissue, control colon tissue and lymph nodes for mRNA analysis and immunohistochemistry/immunomorphometry.

Type of analyzed tissue	Type of analysis	n*	Gender		Age (years)		TNM-stage				Location in colon		
			Male	Female	Median	Range	I	II	III	IV	Right	Transverse	Left
Primary CC tumor	mRNA levels	32	13	19	72	43-86	12	10	8	2	14	1	17
	IHC**	10	5	5	72	60-84	1	3	4	2	6	2	2
Normal colon°	mRNA levels	30	17	13	72	57-85	7	17	4	2	16	2	12
	IHC	9	4	5	70	41-83	1	3	2	3	6	1	2
H&E(+) and H&E(-) lymph nodes of CC patients	mRNA levels	28	11	17	76	50-88	5	8	11	6^#^	16	4	10
	IHC	10	2	8	80	71-91	1	2	7	0	7	1	2
Lymph nodes of control patients^§^	mRNA levels	10	7	3	22	9-24	–^&^	–	–	–	–	–	–

*n = number of patients who had donated samples of normal colon tissue, primary CC tumor tissue and lymph nodes, respectively.

**IHC = Immunohistochemistry and immunomorphometry.

°Distal resection margins of primary CC tumors.

^#^Two TNM-stage IV patients donated 2 lymph nodes, i.e., 6 nodes analyzed from 4 patients.

^§^mRNA expression levels were determined in one lymph node per patient from 8 patients with ulcerative colitis, 1 patient with Crohn’s colitis and 1 patient with lipoma.

^&^– = not applicable.

### Patients and tissue specimens for immunohistochemistry

Primary tumor tissue specimens were from 10 CC patients. None of the patients received treatment before surgery. Normal colon tissue samples were from the distal resection margin of 9 CC patients. Fresh colon tissue samples were snap-frozen and stored at –70°C until preparation of tissue sections as described elsewhere (26). LNs were from additional 10 CC patients and fresh-frozen in the same way as tumor tissue and stored at –70°C. The clinical characteristics of patients donating tissue for immunohistochemical and immuno-morphometry analysis are summarized in **Table 1**.

### Cell lines

Total RNA from the human CC cell lines LS174T, HT29, T84, HCT8, CaCo2, the T-cell line Jurkat, a mixture of the B-cell lines CNB6 and KR4, the monocyte cell line U937, the granulocyte cell line HL60, and the primary foreskin fibroblast cell line FSU, were used and handled as described earlier (27).

### RNA preparation and real-time quantitative reverse transcriptase-polymerase chain reaction (qRT–PCR)

Total RNA was extracted using the acid guanidine phenol chloroform method as described (4). Quantification of FOXQ1, MMP11, THBS2 and CXCL12 mRNAs was performed using commercially available TaqMan: Gene Expression Assays (FOXQ1: Hs00536425_s1), (MMP11 Hs00968295_m1), (THBS2: Hs01568063_m1) and (CXCL12: Hs00930455_m1) in combination with TaqMan EZ technology (Applied Biosystems, Foster City, CA, USA). The RT-PCR profile was 50°C for 2 min, 60°C for 30 min and 95°C for 5 min followed by 45 cycles of 95°C for 20 s and 60°C for 1min. Assays for CXCL16 and CXCL17 are previously described (8, 9). The concentration of 18S rRNA was determined in each sample by using a commercial TaqMan, real-time qRT–PCR assay (Applied Biosystems) for normalization of mRNA levels as described (28). All qRT-PCR analyses were performed in triplicates. Emission from the released reporter dye was recorded by the QuantStudio 5 Real-Time PCR System (Applied Biosystems) or the ABI Prism 7900 HT Sequence Detection System (Applied Biosystems). mRNA levels are expressed as relative quantity (RQ) where the RNA concentrations are normalized to the 18S rRNA concentration in the same sample by calculating the ΔCT between the CT for the mRNA species and the CT for 18S rRNA and RQ calculated according to the equation: 2^- (ΔCT of the sample – the median ΔCT-value of the respective control samples as indicated) (8).

### Antibodies and substrate

The following primary antibodies were used: mouse monoclonal antibody (mAb) anti-FOXQ1 (LS-B6409, Nordic Biolabs, Täby, Sweden), mouse mAb anti-MMP11 (NBP1-40030, Novus, Littleton, CO, USA), rabbit polyclonal IgG anti-THSB2 (ab84469, Abcam, Cambridge, MA, USA), anti-carcinoembryonic antigen (CEA) mAb (IgG1, clone II-7, Dako, Glostrup, Denmark). Mouse IgG, ready to use (Dako) and rabbit IgG ready to use (Dako) substituted primary antibody in negative controls. ImmPRESS micropolymer horse radish peroxidase (HRP) conjugated anti-rabbit IgG (MP-7401, Vector laboratories, Burlingame, CA, USA), ImmPRESS micropolymer HRP conjugated anti-mouse IgG (MP-7402, Vector laboratories), were used as secondary antibodies. The peroxidase substrate was 3,3′-diaminobenzidine (DAB; Vector Laboratories).

### Immunohistochemistry

Frozen tissue was cut into 4 – 6 μm-thick sections and processed as described (27, 29). Briefly, consecutive sections were fixed with 4% paraformaldehyde, endogenous peroxidase activity inhibited by incubation with 0.03% H_2_O_2_ and 2 mM NaN_3_, and non-specific binding sites blocked by incubation with 0.2% bovine serum albumin and 2.5% horse serum (ImmPress, Vector Laboratories). Subsequently, the sections were incubated with primary antibody followed by incubation with ImmPress anti-mouse IgG or ImmPress anti-rabbit IgG, respectively. Bound peroxidase was revealed by incubation with 0.05% DAB and 0.03% H_2_O_2_ in 0.05 M Tris-buffer (pH 7.6), and sections counterstained with methyl green. Anti-CEA mAb and mouse IgG or rabbit IgG instead of primary antibody served as positive and negative control, respectively.

### Immuno-morphometry

The number of positive cells in immunohistochemically stained tissue sections was quantified according to Weibel (27, 30), analyzing the expression of the three fibroblast markers; FOXQ1, MMP11 and THBS2 in tumor cells and in the surrounding stroma in comparison to controls. Twenty randomly chosen ocular fields were counted for each marker in each compartment. One observer (HA) performed the analyses and the slides were coded to avoid personal bias. An integrating cooled color 3CCD camera (Colour Chilled 3 CCD Hamamatsu CameraC5810; Hamamatsu Photonics, Hamamatsu City, Japan) was used on a standard light microscope combined with a computer image analysis system (LeicaQWin, Leica Imaging Systems, Cambridge, UK) with an interactive, computerized morphometry program, as described (31, 32). Twenty microscopic fields were selected randomly using 40X objective and transferred to the screen, onto which a regular 121-points grid was superimposed. Points outside the concerned tissue compartment and empty spaces were not included in the calculation. Positive cells located in the coarse points were counted, and the ratio between the number of points covering positive cells and the total number of points covering the tissue under investigation was calculated for each microscopic field. The mean value was used as estimate of numbers of positive cells for each patient.

### Statistical analysis

The statistical significance of differences in mRNA levels and in numbers of positive cells were calculated using the two-tailed Mann–Whitney rank sum test. Correlations between mRNA levels were analyzed using the non-parametric two-tailed Spearman rank correlation. Descriptive values of mRNA levels are given as median with interquartile range (IQR), while descriptive values of numbers of positive cells by immunomorphometry are given as mean ± 1 standard error of the mean (SEM). The software utilized for statistical calculations was GraphPad Prism 6 (Graphpad Software, San Diego). A *P*-value ≤ 0.05 was considered statistically significant.

### Ethical considerations

Tissue samples were obtained after patient’s consent. The Local Ethics Research Committee of the Medical Faculty, Umeå University approved this study (registration number 03-503; date of approval 3 December 2003).

## Results

### mRNA expression levels of FOXQ1, MMP11, THBS2 and CXCL12 in human cell lines

**Figure 1:**
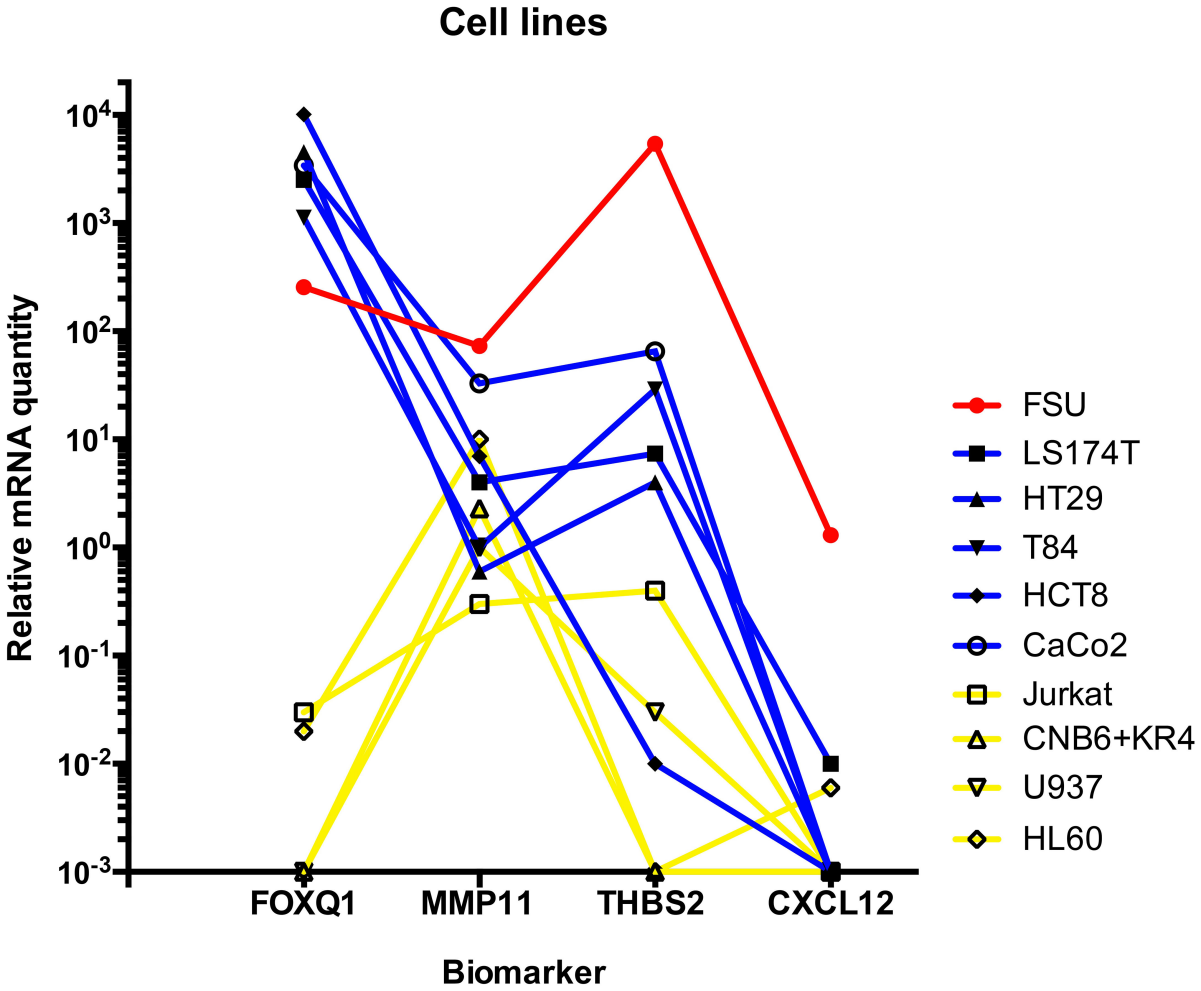
Levels of FOXQ1, MMP11, THBS2, and CXCL12 mRNAs in fibroblast cell line FSU(red line), colon carcinoma cell lines LS174T, HT29, T84, HCT8, and CaCo2 (blue lines) and immune cell lines Jurkat (T cells), CNB6+KR4 (B cells), U937 (monocytes) and HL60 (granulocytes). Immune cells are connected by yellow lines. mRNA levels are given as relative quantity (RQ) calculated as described in the Materials and Methods section using the median value of 30 normal colon samples as base line with the value 1.0.

The mRNA expression levels of the four biomarkers in one fibroblast cell line, five CC cell lines and four immune cell lines are shown in **Figure 1**. Levels are given as relative quantity using the median value of each biomarker for 30 normal colon samples as 1.0. All four biomarkers were expressed in the fibroblast cell line. FOXQ1, MMP11 and THBS2 but not CXCL12 were expressed in CC cell lines. However, HCT8 expressed THBS2 at a very low level. FOXQ1 and THBS2 were not at all or only weakly expressed in immune cell lines. In contrast, MMP11 was expressed at relatively high levels in the granulocyte- and B cell lines.

### mRNA levels of the biomarkers in primary CC tumors and regional LNs of CC patients and their respective control tissues

**Figure 2:**
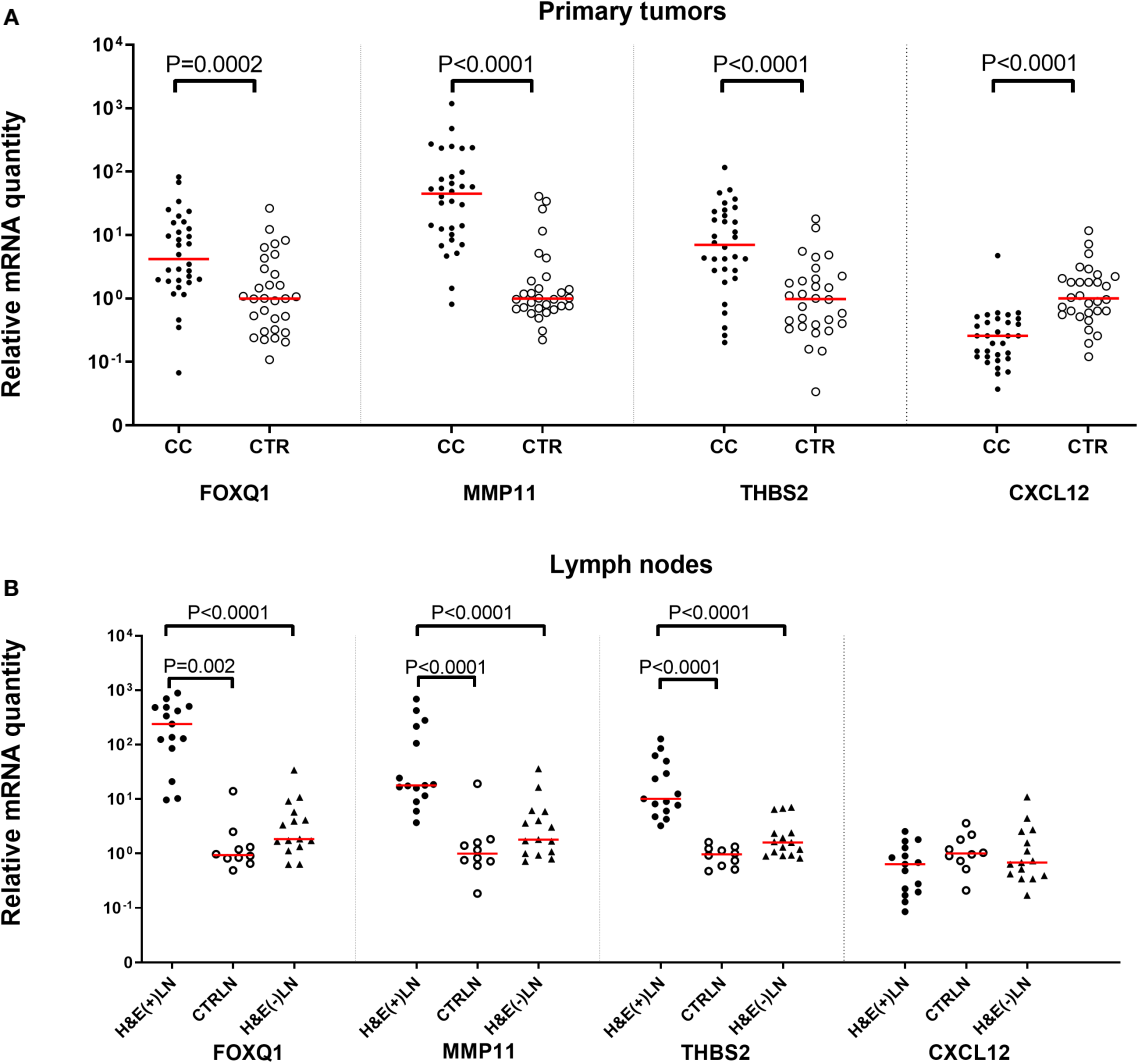
Levels of FOXQ1, MMP11, THB2, and CXCL12 mRNAs in **(A)** primary tumor tissue of 32 CC patients (CC) compared to normal colon tissue (CTR; n = 30) and **(B)** in metastatic lymph nodes of CC patients (H&E(+)LN; n = 15), non-metastatic nodes of CC patients (H&E(-)LN; n = 15) and nodes of control patients (CTRLN; n = 10). mRNA levels are given as relative quantity (RQ) calculated as described in the Materials and Methods section using the median value of 30 normal colon samples as base line with the value 1.0 for primary tumors and normal colon tissue in **(A)** and the median of the 10 CTRLNs in the LN analyses in **(B)**. Red horizontal lines indicate median values. *P*-values were calculated by two-tailed Mann-Whitney rank sum test.

**Figure 2A** shows the mRNA levels of FOXQ1, MMP11, THBS2 and CXCL12 in primary tumors from 32 CC patients compared to normal colon tissue. Relative quantity is calculated in comparison to the medians of normal colon, which are set as 1.0. FOXQ1, MMP11 and THBS2 were expressed at significantly higher levels in primary tumor compared to normal colon (*P*=0.002, *P* <0.0001 and *P* <0.0001, respectively) in spite of the heterogeneity of the control group. The differences between the median levels in the two groups were 6- to 50-fold. In contrast, CXCL12 mRNA levels were higher in normal colon tissue compared to primary tumors (*P*<0.0001).

**Figure 2B** shows the levels of the biomarker mRNAs in metastasis-positive LNs [H&E(+)LN] and metastasis-negative LNs [H&E(-)LN] of CC patients and in LNs of control patients with inflammatory bowel disease. Relative quantity is calculated in comparison to the medians of control LNs, which are set as 1.0. FOXQ1, MMP11 and THBS2 levels were significantly higher in H&E(+)LNs of CC patients compared to control nodes (*P*=0.002, *P* <0.0001 and *P* <0.0001, respectively). The difference between the two groups varied from 9 to 150 times. There was also a highly significant difference in expression levels between H&E(+)LNs compared to H&E(-)LNs (*P*<0.0001) for FOXQ1, MMP11 and THBS2. Moreover, the mRNA levels of the markers FOXQ1, MMP11 and THBS2 were significantly higher in LNs of CC patients with stage III and IV (n = 15) compared to LNs of patients with stage I and II (n = 13) with *P* -values of <0.0001, <0.0001 and 0.0003, respectively. No significant difference between LN-groups was seen for CXCL12.

### Protein level expression of FOXQ1, MMP11 and THBS2, in primary CC tumors, normal colon tissue and LNs of CC patients and controls

**Figure 3:**
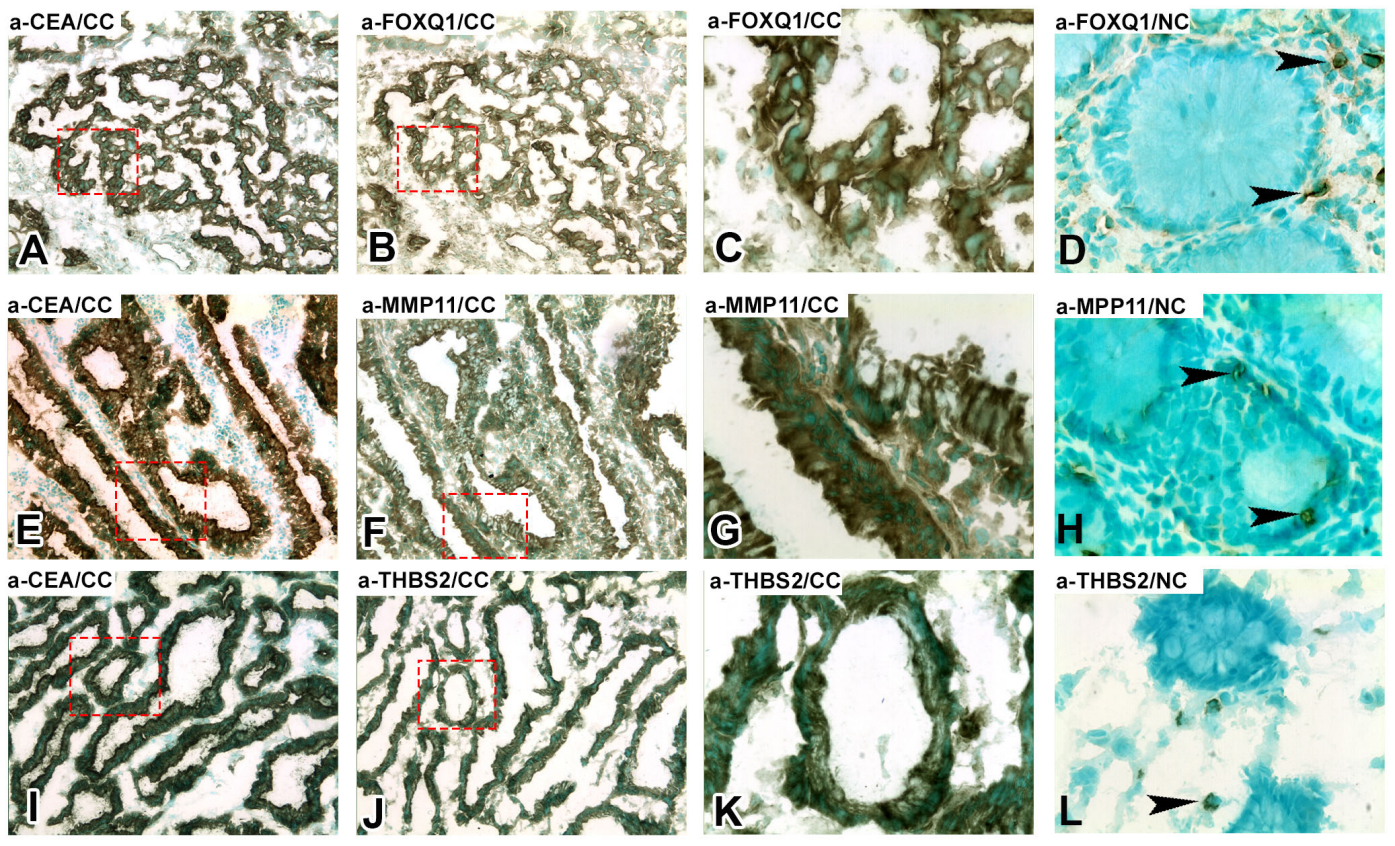
Immunoperoxidase stained tissue sections of a primary tumor (CC) in **(A–C, E–G, I–K)** and normal colon tissue (NC) in **(D, H, L)**. **(A, E, I)**, staining with anti-CEA mAb, 100×. **(B)** Consecutive section of **(A)** stained with anti-FOXQ1 mAb, 100×. **(C)** Higher magnification of the area indicated by hatched red line in **(A, B)**, 400x. **(D)** Normal colon stained with anti-FOXQ1 mAb, 400×, black arrow heads indicate positive stromal cells. **(F)** Consecutive section of **(E)** stained with anti-MMP11 mAb, 100×. **(G)** Higher magnification of the area indicated by hatched red line in **(E, F)**, 400x. **(H)** Normal colon stained with anti-MMP11 mAb, 400×, black arrow heads indicate positive epithelial cells. **(J)** Consecutive section of **(I)** stained with anti-THBS2 rabbit IgG, 100×. **(K)** Higher magnification of the area indicated by hatched red line in **(I)** and **(J)**, 400x. **(L)** Normal colon stained with anti-THBS2 rabbit IgG, 400×, black arrow heads indicate positive stromal cells. Brown to black stained cells indicated positive reaction. Sections were counterstained using methyl-green.

**Figure 4:**
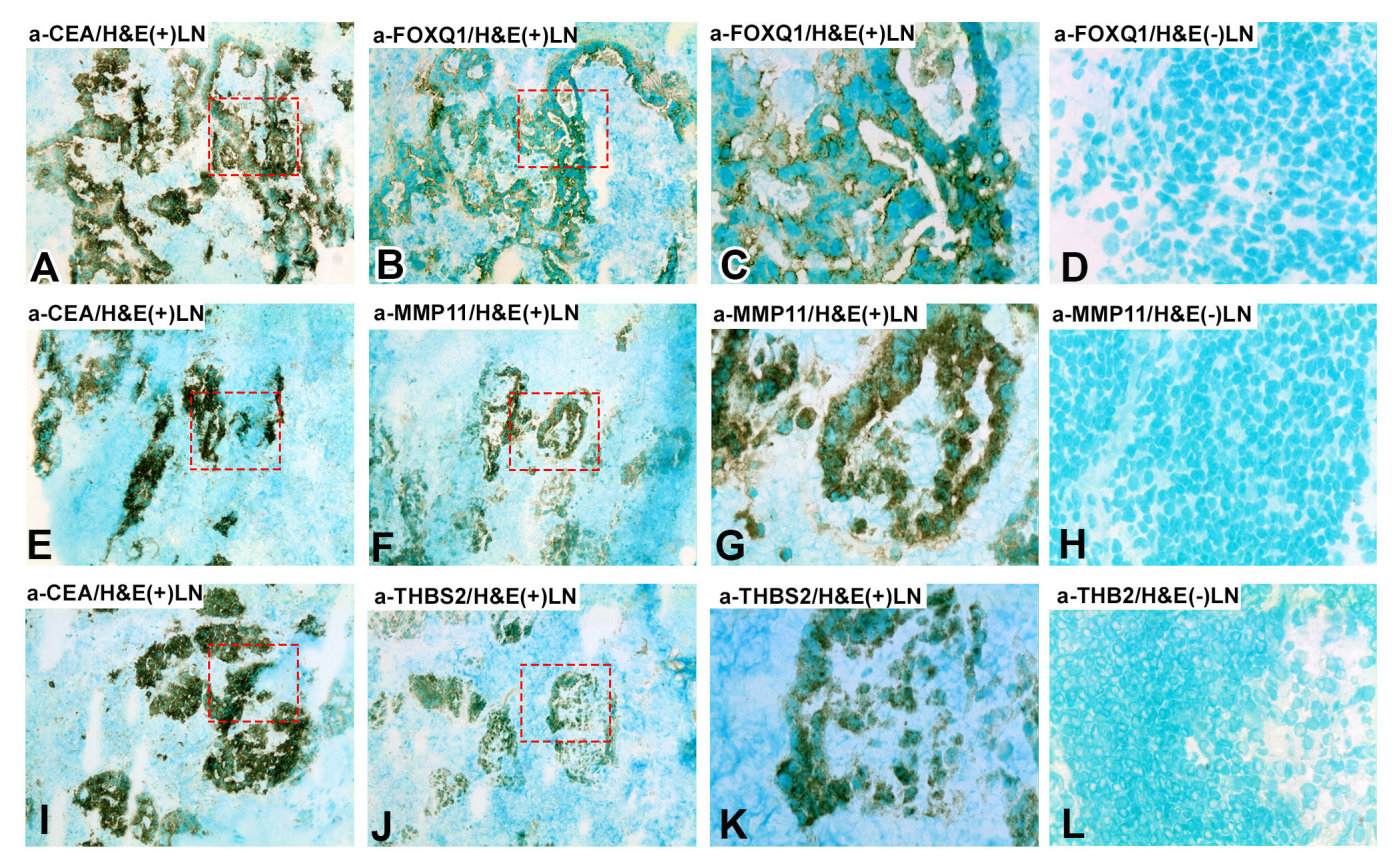
Immunoperoxidase stained tissue sections of lymph nodes of a CC patient. **(A–C, E–G, I–K)** shows a metastasis-positive lymph node [H&E(+)LN] and **(D, H, L)** a metastasis-negative lymph node [H&E(-)LN]. **(A, E, I)**, staining with anti-CEA mAb, 100×. **(B)** Consecutive section of **(A)** stained with anti-FOXQ1 mAb, 100×. **(C)** Higher magnification of the area indicated by hatched red line in **(A)** and **(B)**, 400x. **(D)** Metastasis-negative lymph node of a CC patient stained with anti-FOXQ1 mAb, 400×. **(F)** Consecutive section of **(E)** stained with anti-MMP11 mAb, 100×. **(G)** Higher magnification of the area indicated by hatched red line in **(E, F)**, 400x. **(H)** Metastasis-negative lymph node of a CC patient stained with anti-MMP11 mAb, 400×. **(J)** Consecutive section of **(I)** stained with anti-THBS2 rabbit IgG, 100×. **(K)** Higher magnification of the area indicated by hatched red line in **(I, J)**, 400x. **(L)** Metastasis-negative lymph node of a CC patient stained with anti-THBS2 rabbit IgG, 400×. Brown to black stained cells indicated positive reaction. Sections were counterstained using methyl-green.

The consecutive-sections-immune-staining technique was used to reveal protein expression of FOXQ1, MMP11, and THBS2 in primary CC-tumors and normal colon tissue (**Figure 3**) and in H&E(+)LNs and H&E(-)LNs of CC patients (**Figure 4**). Anti-CEA/CEACAM5 mAb was used to identify the tumor cells in the tissue sections. The staining patterns with antibodies to each of the three biomarkers showed the presence in CC tumor cells and some stromal cells both in primary CC tumors and H&E(+)LNs. Staining appears to be membranous to cytoplasmic. FOXQ1 and THBS2 were barely expressed in normal control colon tissues but occasional stromal cells were stained (**Figures 3D, L**). In contrast, MMP11 positive cells were seen in the epithelium of normal colon tissue (indicated by arrow heads in **Figure 3H**). Very few positive cells were seen in H&E(-)LNs with antibodies to all three biomarkers (**Figures 4D, H, L**).

**Figure 5:**
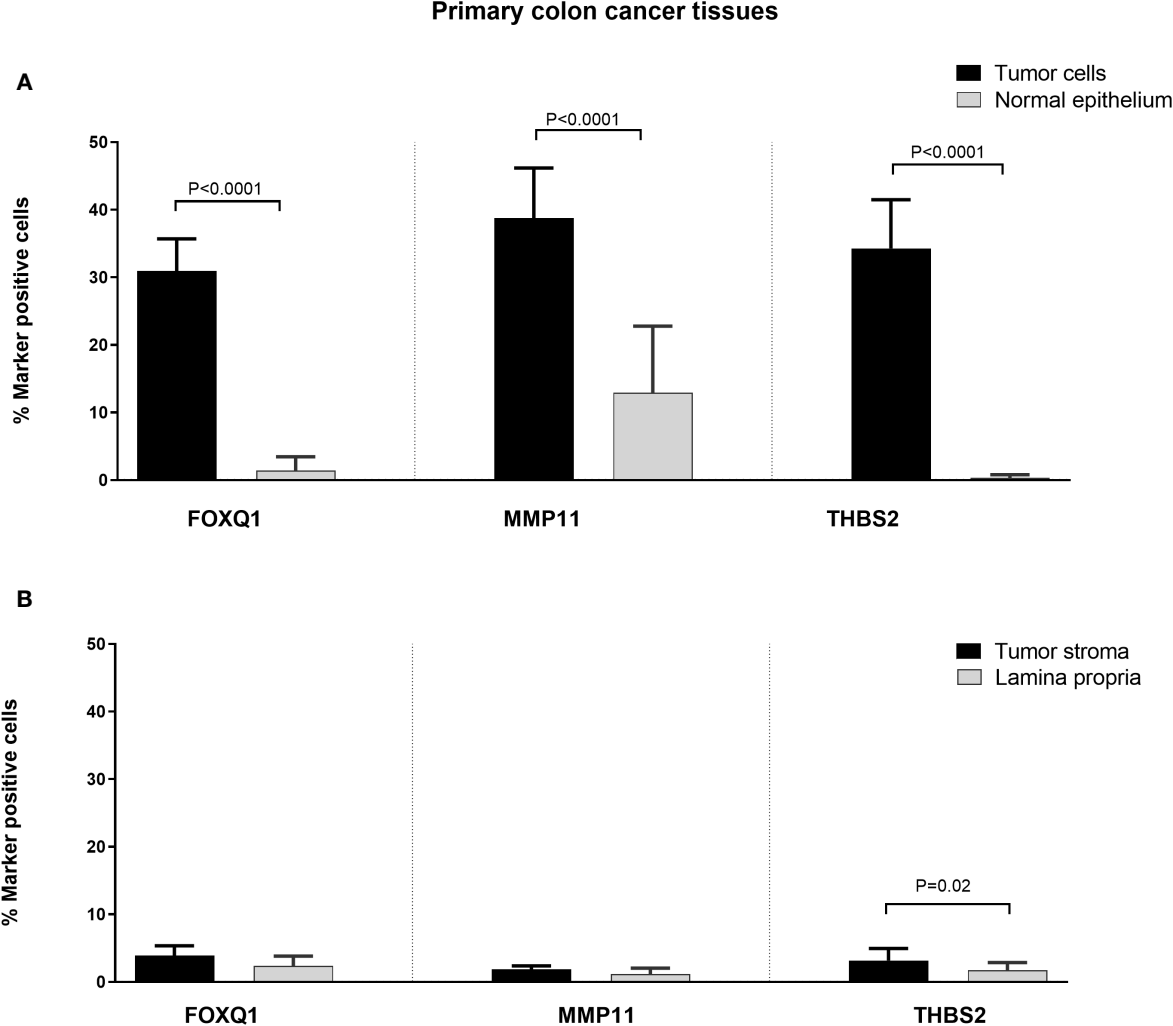
Frequencies of FOXQ1, MMP11 and THBS2 positively stained cells in CC primary tumor tissue compared to normal colon tissue as counted by immunomorphometry analysis according to Weibel. **(A)** CC tumor cells (black bars) compared with normal colon epithelial cells (grey bars). **(B)** CC tumor stroma (black bars) compared with lamina propria in normal colon (grey bars). Frequencies were determined individually in primary tumors of 10 CC patients and normal colon tissue from 9 patients. Bars represent mean+1 SEM. *P*-values for comparisons between tumor and normal tissue by two-sided Mann–Whitney t-test are given.

Immunomorphometry analyses revealed that FOXQ1-, MMP11- and THBS2-positive cells were present at significantly higher numbers in the CC tumor region than in normal colon epithelium (*P* < 0.0001; **Figure 5**). However, the number of MMP11 positive colon epithelial cells was relatively high (mean 12%; **Figure 5**). Between 30–40% of the tumor cells and between 1-3% of the tumor stromal cells were marker-positive with the antibodies against all three biomarkers. Comparing tumor stroma with lamina propria of normal colon mucosa no difference was seen for FOXQ1 or MMP11. However, for THBS2 there was a small but statistically significant difference (*P* = 0.02).

### Correlation between mRNA expression levels of FOXQ1, MMP11 and THBS2 and levels of the ectopically expressed chemokines CXCL17 and CXCL16 in primary CC tumors

**Figure 6:**
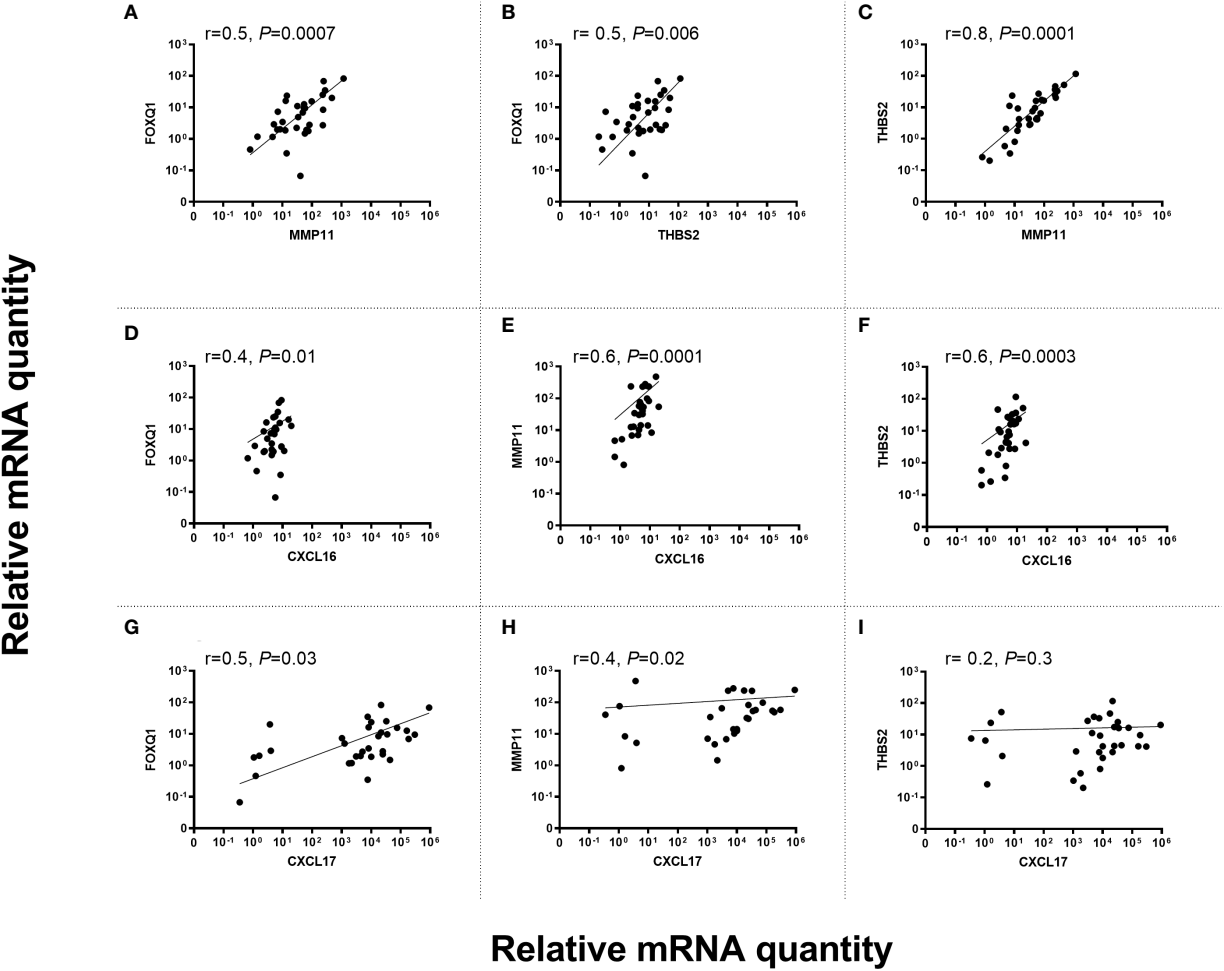
Correlation between mRNA levels of FOXQ1 versus MMP11 **(A)**, FOXQ1 versus THBS2 **(B)**, THBS2 versus MMP11 **(C)**, FOXQ1 versus CXCL16 **(D)**, MMP11 versus CXCL16 **(E)**, THBS2 versus CXCL16 **(F)**, FOXQ1 versus CXCL17 **(G)**, MMP11 versus CXCL17 **(H)** TBHS2 versus CXCL17 **(I)** in primary CC tumors. mRNA levels are given as relative quantity (RQ) calculated as described in the Materials and Methods section. Correlation was calculated using two-sided Spearman rank correlation test.

Potential correlation between levels of the fibroblast biomarkers FOXQ1, MMP11 and THBS2 in primary tumor tissue was investigated. Since FOXQ1 and THBS2 were ectopically expressed in CC tumor cells it was of interest to compare their expression with other ectopically expressed biomarkers like the chemokines CXCL16 and CXCL17 particularly since these two chemokines were associated with bad prognosis (8, 9, 33). **Figure 6** shows the results of the correlation analyses. The results indicate 1) that THBS2 and MMP11 are closely related to each other and that FOXQ1 is more distantly related to them (**Figures 6A–C**), 2) that CXCL16 showed the highest degree of relatedness to THBS2 and MMP11 (**Figures 6E, F**), and 3) that THBS2 expression was completely unrelated to CXCL17 expression (**Figure 6I**).

## Discussion

The most important finding in this study is that the expression levels of the fibroblast biomarkers FOXQ1, MMP11 and THBS2 are significantly elevated in both primary tumor tissue and H&E(+)LNs of CC patients. The increased expression of FOXQ1, and THBS2 is explained by ectopic expression in CC-tumor cells. Thus, while virtually no staining was detected in the normal colon epithelium, as many as 30 to 40% of the tumor cells of the primary tumor stained positively. This is a high number compared to the ectopically expressed chemokines CXCL16 and CXCL17 that were previously shown to stain 5 and 17% of the tumor cells in primary CC tumors (8, 9). Further support for ectopic expression of these two biomarkers was obtained by the demonstration of their mRNAs in CC cell lines, 5/5 for FOXQ1 and 4/5 for THBS2. The Human Protein Atlas confirms expression of FOXQ1, but not THBS2, mRNA in human CC cell lines (7). In contrast, the high level of expression of MMP11 protein in CC tumor cells is not, or only partly, explained by ectopic expression since the level in normal colon epithelium was as high as 12%. MMP11 mRNA was expressed in 5/5 CC cell lines, confirming the results in the Human Protein Atlas and those of Barassa et al., who demonstrated expression in 3/3 adenocarcinoma cell lines (7, 34).

It is not possible to compare expression levels of different biomarkers using relative quantity. However, the levels of FOXQ1 were more than 4-fold higher in CC cell lines compared to fibroblasts while levels of MMP11 and THBS2 were lower in CC cell lines than in fibroblasts. The fact that FOXQ1 and THBS2 are not expressed or expressed at very low levels in immune cells should make these two markers suitable for LN analysis, which has proved to be superior to analysis of primary tumor for prediction of outcome by molecular techniques (4, 35). Previously we have shown that another fibroblast marker, POSTN, is associated with poor prognosis (36). POSTN is, however, not expressed in the tumor cells. Still, elevated POSTN mRNA levels in LNs from CC patients is an important contributor in ColoNode assay being one of five biomarkers in the two-triplex assay for prediction of tumor recurrence in CC (37).

It is interesting to note that not only activated fibroblast promote tumor growth, but the tumor cells themselves appear to be able to take over properties that are linked to providing supportive functions by the microenvironment, in this case functions linked to activated fibroblasts, making tumor cells self-supportive. It could be speculated that among the heterogenous group of tumor cells that leave the primary site those that have acquired several of these properties would be the most aggressive giving rise to distant metastases. Therefore, it would be of interest to determine if there is a fraction of the cells that have acquired two of more of these ectopically expressed molecules.

Both FOXQ1 and THBS2 have promotional effects on CRC cell progression partly by modulating the Wnt/β-catenin and the HB-EGF/EGFR signaling pathways, thus targeting these molecules may have inhibitory effect on CRC (10, 12, 20). It is not yet known whether high levels of FOXQ1, MMP11 and/or THBS2 are indicators of poor prognosis. It is, however, interesting to note that their levels were significantly higher in LNs of stage III/IV patients compared to LNs of stage I/II patients consistent with the idea that high levels in LNs is associated with poor outcome. We consider FOXQ1 and THBS2 as the most promising candidates to predict outcome and would choose them for further studies in a larger clinical material of LNs of CRC patients. We found that FOXQ1 was expressed at high levels in CC cell lines, primary CC tumor tissue and H&E(+)LNs of CC patients. FOXQ1 was reported to be expressed in many epithelial cell lines and to be associated with epithelial to mesenchymal transition in breast cancer making it a good candidate for studies also in CRC (38, 39). However, previous studies on the prognostic value of FOXQ1 in primary tumor are conflicting. One study judged FOXQ1 not to be a useful prognostic marker in CRC when it was investigated for predicting outcome, thus, if elevated in the primary CRC tumor there was a 5-years survival in the high group of 52% compared to 66% in the low group (*P*=0.11) (7). The other study identified FOXQ1 as a promising prognostic biomarker in CRC and supported the finding with knock-down experiments (40). To our knowledge there are no reports on the prognostic value of FOXQ1 when analyzed in LNs, which according to our experience can be very rewarding in cases were analysis of the primary tumor only shows a trend. THBS2 was found to have low mRNA expression levels in immune cells and LNs of control patients and at the protein level there was a very clear distinction between primary tumor and normal colon tissue. THBS2, is an adhesive glycoprotein that mediates cell-to-cell and cell to-matrix interactions, suggesting a role in escape and dissemination of the tumor cells (41).

Future studies will show whether these ectopically expressed fibroblast biomarkers have a prognostic value, alone or in combination with other biomarkers.

## Data availability statement

The original contributions presented in the study are included in the article. Further inquiries can be directed to the corresponding author.

## Ethics statement

The studies involving humans were approved by The Local Ethics Research Committee of the Medical Faculty, Umeå University (registration number 03-503; date of approval December 3, 2003). The studies were conducted in accordance with the local legislation and institutional requirements. The participants provided their written informed consent to participate in this study.

## Author contributions

HA: Data curation, Formal Analysis, Methodology, Software, Writing – review & editing. MA: Data curation, Methodology, Writing – review & editing. LO: Methodology, Writing – review & editing. GL: Conceptualization, Formal Analysis, Writing – review & editing. M-LH: Conceptualization, Data curation, Funding acquisition, Writing – original draft, Writing – review & editing. SH: Conceptualization, Data curation, Writing – original draft, Writing – review & editing. BS: Conceptualization, Funding acquisition, Methodology, Project administration, Writing – original draft, Writing – review & editing.
